# Thermal Conductivity Analysis of Chitin and Deacetylated-Chitin Nanofiber Films under Dry Conditions

**DOI:** 10.3390/nano11030658

**Published:** 2021-03-08

**Authors:** Jiahao Wang, Keitaro Kasuya, Hirotaka Koga, Masaya Nogi, Kojiro Uetani

**Affiliations:** 1 Graduate School of Engineering, Osaka University, Mihogaoka 8-1, Ibaraki-shi, Osaka 567-0047, Japan; w541725924@eco.sanken.osaka-u.ac.jp (J.W.); keitaro_k@chem.eng.osaka-u.ac.jp (K.K.); 2 The Institute of Scientific and Industrial Research (SANKEN), Osaka University, Mihogaoka 8-1, Ibaraki-shi, Osaka 567-0047, Japan; hkoga@eco.sanken.osaka-u.ac.jp (H.K.); nogi@eco.sanken.osaka-u.ac.jp (M.N.)

**Keywords:** chitin nanofiber, deacetylated chitin, nanopaper, thermal diffusivity

## Abstract

Chitin, a natural polysaccharide polymer, forms highly crystalline nanofibers and is expected to have sophisticated engineering applications. In particular, for development of next-generation heat-transfer and heat-insulating materials, analysis of the thermal conductivity is important, but the thermal conductivity properties of chitin nanofiber materials have not been reported. The thermal conductivity properties of chitin nanofiber materials are difficult to elucidate without excluding the effect of adsorbed water and analyzing the influence of surface amino groups. In this study, we aimed to accurately evaluate the thermal conductivity properties of chitin nanofiber films by changing the content of surface amino groups and measuring the thermal diffusivity under dry conditions. Chitin and deacetylated-chitin nanofiber films with surface deacetylation of 5.8% and 25.1% showed in-plane thermal conductivity of 0.82 and 0.73 W/mK, respectively. Taking into account that the films had similar crystalline structures and almost the same moisture contents, the difference in the thermal conductivity was concluded to only depend on the amino group content on the fiber surfaces. Our methodology for measuring the thermal diffusivity under conditioned humidity will pave the way for more accurate analysis of the thermal conductivity performance of hydrophilic materials.

## 1. Introduction

Chitin is a linear crystalline polymer consisting of *N*-acetylglucosamine with *β*-(1→4) linkages that is widely produced by insects [1], fungi [2,3], and crustaceans [4]. The crystalline chitin nanofibers extracted from native chitins have uses as engineering materials, such as high-strength composites [5], low-thermal expansion and transparent films [6,7], electronics [8], microparticles [9], membranes [10], and chiral nematic mesoporous materials [11], as well as in biomedical applications [12]. In particular, chitin nanofibers have been reported to have extremely high strength owing to their high crystallinity [13], and there are high expectations for their use in engineering [14,15]. Recently, it has been reported that highly crystalline cellulose nanofibers have high thermal conductivity compared with plastics and glass [16], and unique thermal application pathways have been developed, such as transparent and thermal conductive films [17], directional heat flow films [18,19], and heat-transfer modulation films [20]. Nanofibers of chitin, a similar polysaccharide to cellulose with high crystallinity, are expected to have similar properties, but thermal conductivity analysis of chitin nanofiber materials has not been performed.

Clarification of the thermal conductivity properties of chitin nanofiber materials toward unexplored thermal applications remains a major challenge. In analyzing the thermal conductivity properties of chitin nanofiber materials, there are three inherent problems. First, natural chitin has a small content of amino groups (chitosan segments), which results in an irregular chemical structure on the nanofiber surface. It is, therefore, important to evaluate the effect of the different content of amino groups on the thermal conductivity. Second, chitin nanofibers have a highly anisotropic fibrous morphology, and when they are dried as a nonwoven film, there are large structural differences between the film’s in-plane and through-plane directions. It is necessary to separately measure the thermal conductivity properties for each direction. Third, because chitin nanofibers are hydrophilic, like cellulose, moisture adsorption will occur when the nanofiber films are investigated in the ambient environment. This moisture adsorption is known to have a strong tendency to swell and irregularly bend the films. To accurately analyze the thermal conductivity of the chitin nanofiber film, it is necessary to measure the thermal conductivity while keeping the film as dry as possible.

In this study, we aimed to elucidate the thermal conductivity properties of chitin nanofiber films by technically addressing the above three issues. For the first issue, partially deacetylated samples were prepared by alkaline treatment to clearly increase the content of surface amino groups, according to a previous study [21]. For the second issue, we used the nondestructive and noncontact method of the laser spot periodic heating radiation thermometry method [22], which allows independent measurement of film-like materials in the in-plane and through-plane directions. For the third issue, a specially designed sealed chamber was used to realize the thermal diffusivity measurement in a very low humidity environment.

## 2. Materials and Methods

### 2.1. Materials

A total of 175 g of the leg shell of snow crab (*Chionoecetes opilio*) was boiled for 1.5 h and then cut into ~2 cm × 2 cm square pieces. The shells were then immersed in 2 L of 5 wt% NaOH for 6 h at 90 °C without vigorous stirring. Subsequently, the product was cooled to room temperature and filtered to repeatedly wash with distilled water until the pH reached neutral. The product was then immersed in 2 L of 5 wt% HCl at room temperature for 48 h, followed by filtering to remove minerals and proteins. These alkaline and acid treatments were repeated another two times. For these repetitions, the treatment time was reduced to 2 h. The product was then bleached with 1.5 L of aqueous NaClO_2_ solution at 80–90 °C under acidic conditions, where the pH was adjusted to ~3 by adding acetic acid. We added 15 g of NaClO_2_ and acetic acid per hour for 3 h, followed by repeating for another 3 h with a decreased NaClO_2_ amount of 7.5 g per hour. After complete bleaching, the sample was washed to obtain bleached crab shell.

Wet bleached crab shell (13.6 g, corresponding to dry weight of 3 g) was suspended in 75 mL of 33.6 wt% NaOH solution and mixed with 0.09 g NaBH_4_ at 90 °C for 4 h [21]. NaBH_4_ was added to prevent depolymerization [21,23]. During the reaction, the beaker was shaken every 15–20 min. The product was then repeatedly washed with distilled water to obtain deacetylated crab shell.

The bleached and deacetylated crab shells were used to produce chitin nanofiber (ChNF) and deacetylated-ChNF (D-ChNF), respectively. Both of the crab shells dispersed in 500 mL of distilled water were agitated by a high-speed blender with a 2 L stainless-steel bottle (CAC90B X-TREME, Waring Commercial, Conair Corp., Stamford, CT, USA) combined with an Absolute3 motor (Vitamix, Cleveland, OH, USA) at 24,000 rpm for 12 min for preliminary fibrillation. The suspensions diluted to 1 L were then passed through a high-pressure water-jet system (Starburst 10, Sugino Machine Limited, Toyama, Japan) with a ball-type chamber at 180 MPa for 50 cycles for fibrillation into nanofibers. The final suspensions for ChNF and D-ChNF had concentrations of 0.20 and 0.16 wt%, respectively.

### 2.2. Film Fabrication

The ChNF and D-ChNF suspensions were filtered by a membrane filter (A010A047A, Advantec Toyo Kaisha, Ltd., Tokyo, Japan) to form wet mats. Another membrane filter was attached to the upper surface of the wet mats, and they were then hot-pressed at 110 °C for 15 min to dry the nanofiber films with thickness of ~55 μm.

### 2.3. Degree of Deacetylation

The degree of deacetylation of the nanofiber samples was measured through conductometric titration. The suspensions of the never-dried nanofiber samples were further diluted to 0.12–0.17 wt% with distilled water, and 5 mL of 0.01 M NaCl solution was added. The pH was then adjusted to ~10 by adding 0.05 M NaOH. The suspension was stirred for 30 min, followed by adjusting the pH to ~2.8 by adding 0.1 M HCl. The suspension was then titrated by adding 0.05 M NaOH at a speed of 0.1 mL/min using an automatic titrator (AUT-701, DKK-TOA Corp., Tokyo, Japan).

According to a previous method [24], the degree of deacetylation was calculated using the following equations:

(1) $$n\;\left(\mathrm{mmol}/g\right)\;=\frac{V\;\times \;c}{w}$$

(2) $$\;\mathrm{Degree}\;\mathrm{of}\;\mathrm{deacetylation}\;\left(\%\right)\;=\;\frac{203.1927\;\times \;n/1000}{1\;+\;42.0358\;\times \;n/1000}$$

where *n*, *V*, *c*, and *w* are the amino group content, consumed volume of NaOH solution at the plateau region of the titration profile, concentration of the titrated NaOH, and weight of the measured sample, respectively.

### 2.4. Characterization

X-ray diffractometry was performed on the films prepared in Section 2.2 using a Miniflex600 diffractometer (Rigaku Corp., Tokyo, Japan) with monochromatic Cu K*α* radiation generated at 40 kV and 15 mA. The scattering angle 2*θ* was scanned from 5° to 35° at steps of 0.01°. The crystallite width (*D*) of each diffraction plane (*hkl*) was calculated by the method of full width at half-maximum (fwhm) using Scherrer’s equation: $D_{hkl}\;=\;K\lambda /\beta \cos\theta$, where *K* = 0.9, *λ* is the wavelength (1.541862 Å), *β* is the fwhm of the peak, and *θ* is the Bragg angle. The relative degree of crystallinity *I*_C_ was estimated from the area ratios of the crystalline peaks to the sum of the separated peaks.

Field emission scanning electron microscopy (FESEM, JSM-F100, JEOL Ltd., Tokyo, Japan) observation was performed at an acceleration voltage of 1 kV. The nanofiber films were prepared by casting 5 mL of the suspensions mixed with the same volume of acetone on a Teflon dish, drying in an oven at 55 °C, and then coating with Au by an ion sputterer (E-1045, Hitachi High-Tech Corp., Tokyo, Japan).

Transmission electron microscopy (TEM) observation was performed on two types of nanofibers using a JEM-ARM200F (JEOL, Ltd., Tokyo, Japan) at an acceleration voltage of 200 kV. Samples were negatively stained with the phosphotungstic acid.

The moisture content of the films conditioned under humidity below 10% at 25 °C for more than 1 day was calculated from the weight loss of the film heated at 100 °C for 60 min. After conditioning the film under constant humidity, the film was promptly introduced into a thermogravimetric analyzer (Q50, TA Instruments Japan, Tokyo, Japan) and the temperature was rapidly increased to 100 °C to perform isothermal gravimetry for 60 min under a nitrogen atmosphere. The measurements were performed three times per film.

### 2.5. Thermal Conductivity Measurement under Dry Conditions

The thermal conductivity of the film *κ* was calculated by the equation *κ* = *αρC*_p_, where *α* is the thermal diffusivity, which was measured by the unsteady method, *C*_p_ is the specific heat capacity, and *ρ* is the bulk density. In the following, the subscripts T and I for *κ* and *α* represent the through-plane and in-plane direction, respectively.

The thermal diffusivity was measured by the laser spot periodic heating radiation thermometry method using a TA33 thermowave analyzer (Bethel Co., Ltd., Ibaraki, Japan). Both sides of the film were blackened with graphite spray to prevent permeation of the laser and to keep the emissivity constant in order to ensure the accuracy of temperature detection. A sealing chamber was custom-made to measure the thermal diffusivity of the films under dry conditions. The sample was placed on a stainless-steel washer and then affixed to the sealing with polyimide tape. The sealing chamber holding the sample was conditioned to relative humidity of below 10% in the tightbox with an excess amount of dried silica gel for at least 1 day. After conditioning, the chamber lid was promptly tightened to seal the chamber under the conditioning environment, and the thermal diffusivity was measured. For measurement of the thermal diffusivity in the through-plane direction (*α*_T_), the frequency of the heating laser *f* was set from 10.13 to 60.13 Hz with a step size of 2.0 Hz, and its intensity was fixed at 8%. Conversely, during detection of the thermal diffusivity in the in-plane direction (*α*_I_), *f* was set to 0.16, 0.21, 0.31, 0.41, 0.51, and 0.61 Hz with the intensity of the heating laser set to 12%, and the distance was changed from 0.4 to 0.7 mm, with a step size of 0.03 mm.

The specific heat capacity *C*_p_ was measured by a differential scanning calorimeter (Thermo Plus Evo2 8230, Rigaku, Japan). The sample weight ranged from 4 to 6 mg and the scanning temperature ranged from −30 to 130 °C at a heating rate of 5 °C/min. We measured the second or third heating scan to remove the effect of absorbed moisture [16]. In brief, after heating the sample to 130 °C in the first scan, the sample temperature decreased to 40 °C at a rate of −30 °C/min, and the weight of the sample was remeasured. The sample was then remounted to cool to −30 °C for the second scan. *C*_p_ was calculated from the data of the second or third scan.

The bulk density of the nanofiber film was calculated from its weight and volume. The films were stored in a tightbox in the dry state (relative humidity below 10%) until just before the thickness and weight measurement.

## 3. Results

### 3.1. Characterization of the Nanofiber Samples

**Figure 1:**
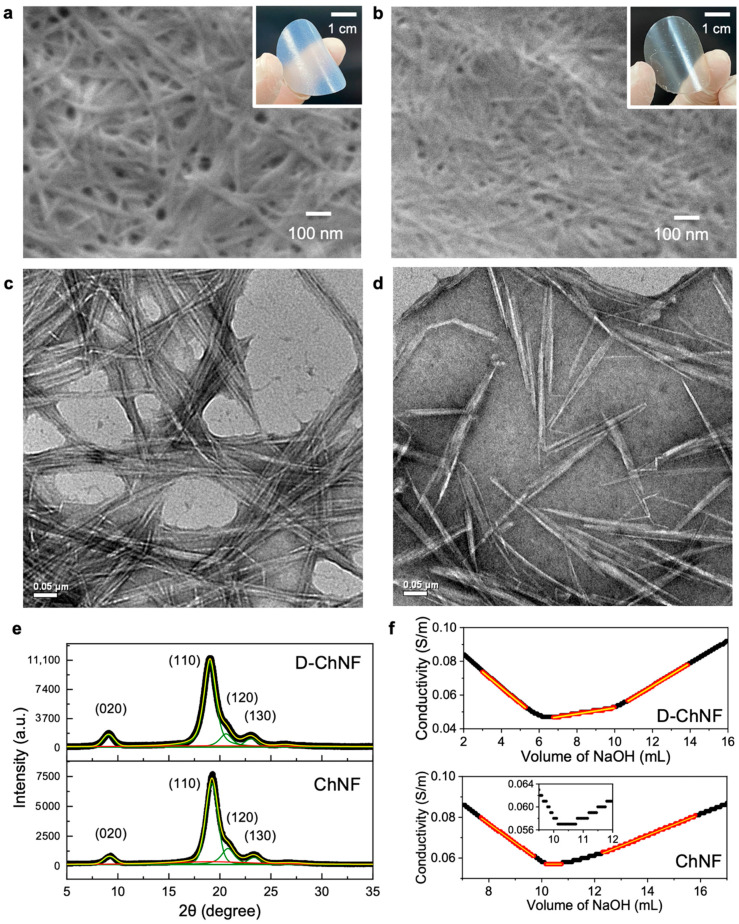
Characterization of D-ChNF and ChNF. FESEM images of (**a**) ChNF and (**b**) D-ChNF. The inserts show the appearance of the films. TEM images of (**c**) ChNF and (**d**) D-ChNF. (**e**) Typical X-ray diffraction profiles of D-ChNF and ChNF. The profiles were deconvoluted into the green and red peaks originating from crystalline and amorphous diffraction, respectively, and fitted by the cumulative fit (yellow profile). (**f**) Conductometric titration plots of D-ChNF and ChNF. The red plots were fitted with the yellow lines, and consumption of 0.05 M NaOH solution was calculated from the two intersections of the three fitted lines. The inset for ChNF shows the magnified plot at around the plateau region.

Both the ChNF and D-ChNF films showed a translucent appearance owing to their fine nanofiber network structures (Figure 1a,b). The apparent transparency is thought to be related to the chemical composition of the nanofiber surface and bulk density, where the bulk densities of the ChNF and D-ChNF films were 0.98 and 1.20 g/cm^3^, respectively. In addition, the ChNF film might have slightly thicker bundles than D-ChNF, even after high-pressure water-jet treatment, which may also explain the smaller packing density and hazy appearance of the ChNF film. High resolution TEM observation (Figure 1c,d) shows that the ChNF tends to be slightly bunched and aggregated, whereas the D-ChNF tends to be well dispersed. Furthermore, the D-ChNF was found to have a short rod-like (needle-like) whisker shape. It was suggested that the strong alkali treatment for deacetylation and the subsequent water-jet fibrillation treatment shortened the fiber length.

The X-ray diffraction profiles of both the ChNF and D-ChNF samples (Figure 1e) showed typical α-chitin crystals [25,26]. After peak deconvolution, we found similar crystallite sizes and degrees of crystallinity for ChNF and D-ChNF, as displayed in Table 1, indicating that the α-chitin crystal structure was retained after harsh alkaline treatment using 33.6 wt% NaOH for 4 h, and only the nanofiber surface was deacetylated. This result is in good agreement with previous reports [21].

The conductometric titration profiles of ChNF and D-ChNF greatly differed (Figure 1f). For the change in the conductivity with titration of 0.05 M NaOH, the first slope at small titrating volumes was because of neutralization of the strong acid (hydrochloric acid), the plateau region in the center was because of neutralization of the targeted weak acid (amino group), and the following ascending slope was because of the increase in the concentration of the alkali. D-ChNF showed a significantly larger plateau region than ChNF, indicating that NaOH consumption by neutralization of amino groups was higher.

To quantify the content of amino groups, the three regions (Figure 1f) were linearly fitted, and the volume of 0.05 M NaOH solution consumed in the plateau region was calculated from the two intersections. The obtained NaOH volume gave the amino group content and degree of deacetylation (Table 2). ChNF contained a small content of inherent amino groups on its surfaces, and D-ChNF contained ~4.5 times greater content of amino groups than ChNF. These results were consistent with previous reports [21,27]. Thus, we succeeded in preparing ChNFs with different surface amino group contents toward investigating the effect of the amino group content on the thermal conductivity properties.

### 3.2. Analysis of the Thermal Conductivity Properties under Dry Conditions

**Figure 2:**
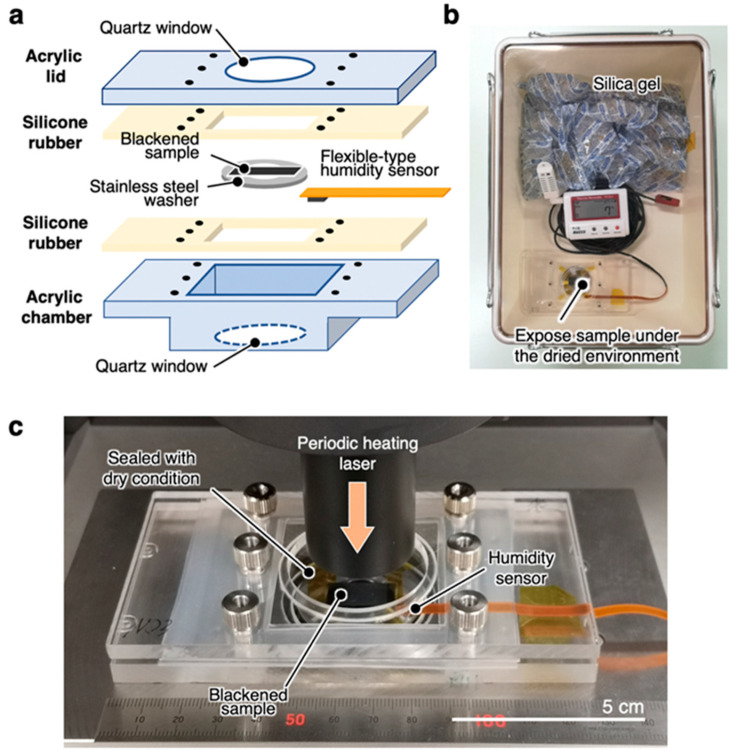
System for measuring the thermal diffusivity under dry conditions. (**a**) Setup of the sealing chamber. (**b**) Chamber holding the sample conditioned in the tightbox under relative humidity below 10% with an excess amount of dried silica gel. (**c**) Sealed chamber under dry conditions mounted on the TA33 stage to measure the thermal diffusivity.

To measure the thermal diffusivity under dry conditions, we used a custom-made sealing chamber that fits the TA33 stage to keep the sample under dry conditions. The samples were fixed in the acrylic sealing chamber shown in Figure 2a and conditioned in a tightbox containing an excess amount of dried silica gel for more than 1 day with the lid open (Figure 2b). The relative humidity in the tightbox was kept below 10%. After sufficient conditioning time, the lid of the sealing chamber was quickly closed, and the thermal diffusivity was measured while maintaining the inside of the chamber under dry conditions (Figure 2c).

The validity of the thermal diffusivity measurements using the chamber was confirmed through measuring a standard sample of a 300-µm-thick copper plate and a cellulose nanofiber film. For all of the samples, the thermal diffusivity values measured with and without the chamber were comparable, and their variation was within the 5% measurement error of the TA33 thermowave analyzer. We concluded that it is possible to accurately measure the thermal diffusivity with a sufficiently small error range using the chamber.

**Figure 3:**
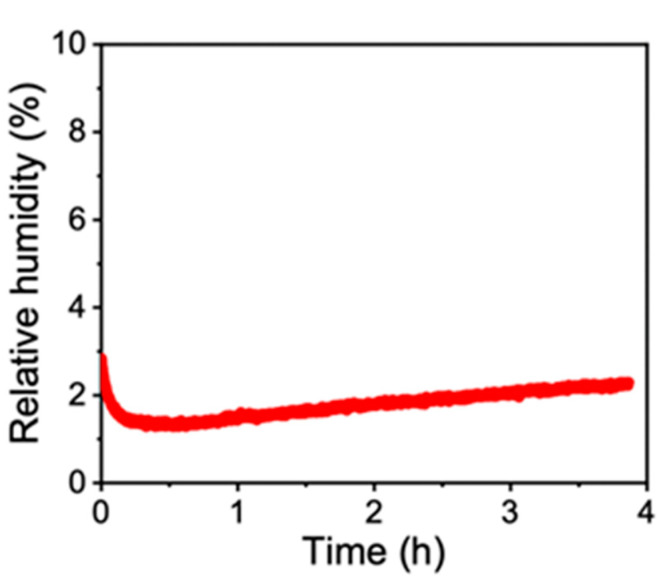
Typical change in the relative humidity in the sealed chamber during thermal diffusivity measurement.

The thermal diffusivities of the ChNF and D-ChNF films were measured under dry conditions. The humidity in the sealed chamber during the measurement was kept constant at 2–3%, as shown in Figure 3, confirming that the measurement was carried out under stable dry conditions owing to sufficient sealing. We waited for 5–15 min before starting the diffusivity measurement to stabilize the humidity inside the chamber.

**Figure 4:**
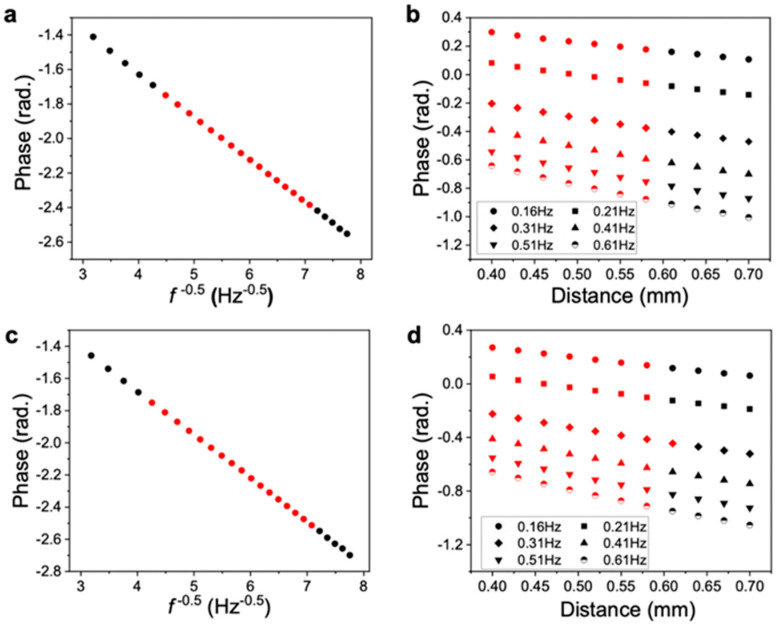
Typical experimental data for measuring the thermal diffusivity. Measurement data of the ChNF film in the (**a**) through-plane and (**b**) in-plane directions. Measurement data of the D-ChNF film in the (**c**) through-plane and (**d**) in-plane directions. The red points were used for linear fitting to determine the slopes.

The measured thermal diffusivity results are shown in Figure 4. In the measurement of the thermal diffusivity in the through-plane direction, the frequency of the periodic heating laser was varied at the same point of heating, and the thermal diffusivity was calculated from the rate of change of the phase measured on the sample backside (slope of the plot). For the in-plane direction, in addition to the heating frequency, the phase detection point on the sample backside needed to be changed, and the diffusivity was calculated from the rate of change of the phase with respect to the distance from the heating point [16,22].

The phase and amplitude of both ChNF and D-ChNF, indicating propagation of the temperature waves, were clearly detected and showed an ideal linear trend (Figure 4), confirming that the measurements were correctly performed. The calculated diffusivities are listed in Table 3. For both films, the thermal conductivity and thermal diffusivity in the through-plane direction were smaller than those in the in-plane direction. This is because of the fiber orientation of the layers within the nonwoven film [16,18]. The in-plane thermal conductivities of both films were in a similar range to those of wood cellulose nanofiber films [16].

While there were no significant differences in the thermal diffusivity and conductivity of the ChNF and D-ChNF films in the through-plane direction, the in-plane thermal diffusivity (*α*_I_) of the D-ChNF film was about 25% lower than that of the ChNF film. The in-plane thermal conductivity of the D-ChNF film was also ~10% lower. The measurement error of the TA33 in measuring the thermal diffusivity is guaranteed to be within ±5%, and we considered the *α*_I_ difference between the films to be significant. This demonstrates that surface deacetylation of ChNF significantly increased the phonon scattering at the nanofiber interface to decrease thermal propagation within the films.

**Figure 5:**
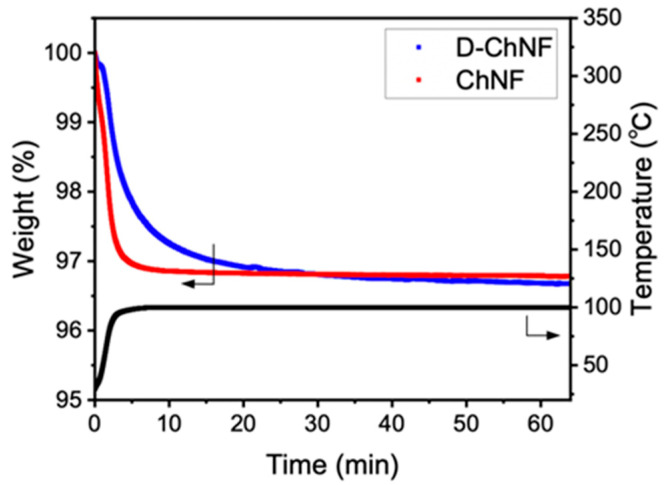
Typical profiles of thermogravimetric analysis of the ChNF and D-ChNF films just after conditioning at relative humidity below 10%.

To investigate the cause of the thermal conductivity difference, the adsorption moisture content of the films was measured. The ChNF and D-ChNF films showed almost the same moisture contents of 3.21% ± 0.31% and 3.32% ± 0.08%, respectively (Figure 5). Thus, even though the contents of surface amino groups differed, the moisture content could be reduced by conditioning the films for a long time in a dry environment with a relative humidity of less than 10%. The dehydration behavior in the thermogravimetric measurements (Figure 5) showed that the moisture desorption rate of D-ChNF was lower than that of ChNF. This appears to be because of the higher surface hydrophilicity of D-ChNF, which is in good agreement with the trend in the content of amino groups. Overall, the difference in the thermal diffusivity detected in this study was determined not by the moisture content or crystal structure, but by the content of surface amino groups.

## 4. Discussion

For nanofiber films of cellulose, a crystalline polysaccharide similar to chitin, the in-plane thermal conductivity is proportional to the crystallite width [16]. In particular, bacterial cellulose nanofiber films with similar crystallite widths to the ChNF film (approximately 5–6 nm) showed in-plane thermal conductivity of about 1.3 W/mK, although the drying conditions were not controlled [16], while the ChNF film showed significantly lower conductivity of 0.82 W/mK. The phonon propagation properties of crystalline nanofiber films were found to differ depending on their molecular composition, as well as the crystallite width.

The thermal diffusivity and thermal conductivity of the D-ChNF film, whose crystal structure and moisture content were almost the same as those of the ChNF film, but containing a larger content of surface amino groups, were clearly reduced compared with the ChNF film. Although the cationic charge increased with the increased content of amino groups, its introduction was partial (only 25%), and the amino groups were thought to be randomly located, because alkaline treatment did not have any site specificity, which may have resulted in an increase of the thermal resistance at the interfiber interface. Since a stronger deacetylation treatment would dissolve chitin, we used the NaOH concentration of ~33 wt%, which is reported not to dissolve chitin [21]. The nanofiber length suggested by the TEM observation is likely to have little effect on the thermal conductivity, according to the previous report [16]. The bulk density of the film is inversely proportional to the in-plane thermal diffusivity [20], so it is reasonable that the D-ChNF film in this study had a higher bulk density and lower in-plane thermal diffusivity than the ChNF film. Although it has been reported that the thermal conductivity of cellulose nanofiber films is modulated by physical perturbation of the fiber–fiber interface [20], further detailed investigation is necessary to enhance the thermal conductivity by chemical modification of nanofiber surfaces.

## 5. Conclusions

In this study, the thermal conductivity of hygroscopic ChNF films was successfully measured under dry conditions. The ChNF and D-ChNF films with surface deacetylation of 5.8% and 25.1% showed in-plane thermal conductivity of 0.82 and 0.73 W/mK, respectively. The films had similar crystalline structures and almost the same moisture content, so the difference in the thermal conductivity was concluded to only depend on the content of amino groups on the fiber surfaces. Our methodology for measuring the thermal diffusivity under conditioned humidity paves the way for more accurate analysis of the thermal conductivity performance of hydrophilic materials.

## Figures and Tables

**Table 1:** Crystallographic analysis.

	*d*_020_	*d*_110_	*d*_120_	*d*_130_	*I*_C_
	nm	nm	nm	nm	%
ChNF	5.87 ± 0.84	5.86 ± 0.11	5.42 ± 1.48	5.67 ± 0.30	83.17
D-ChNF	6.10 ± 0.29	5.85 ± 0.06	5.48 ± 0.42	5.47 ± 0.56	85.54

**Table 2:** Titration analysis to determine the degree of deacetylation.

	*-NH*_2_ *Content*	*Degree of Deacetylation*
	mmol/g	%
ChNF	0.29	5.8
D-ChNF	1.30	25.1

**Table 3:** Thermal conductivity properties of the ChNF and D-ChNF films.

	*ρ*	*C*_p_	*α*_T_	*α*_I_	*κ*_T_	*κ*_I_
	g/cm^3^	J/gK	mm^2^/s	mm^2^/s	W/mK	W/mK
ChNF film	0.98 ± 0.01	1.33 ± 0.06	0.152 ± 0.05	0.625 ± 0.06	0.22 ± 0.06	0.82 ± 0.12
D-ChNF film	1.20 ± 0.03	1.21 ± 0.01	0.135 ± 0.02	0.475 ± 0.01	0.20 ± 0.03	0.73 ± 0.01
